# Generating Diverse Spinal Motor Neuron Subtypes from Human Pluripotent Stem Cells

**DOI:** 10.1155/2016/1036974

**Published:** 2015-12-28

**Authors:** Rickie Patani

**Affiliations:** ^1^Department of Molecular Neuroscience, Institute of Neurology, University College London, London WC1N 3BG, UK; ^2^Department of Clinical Neurosciences, University of Cambridge, Cambridge CB2 0QQ, UK; ^3^University of Edinburgh, Edinburgh EH16 4SB, UK

## Abstract

Resolving the mechanisms underlying human neuronal diversification remains a major challenge in developmental and applied neurobiology. Motor neurons (MNs) represent a diverse pool of neuronal subtypes exhibiting differential vulnerability in different human neurodegenerative diseases, including amyotrophic lateral sclerosis (ALS) and spinal muscular atrophy (SMA). The ability to predictably manipulate MN subtype lineage restriction from human pluripotent stem cells (PSCs) will form the essential basis to establishing accurate, clinically relevant *in vitro* disease models. I first overview motor neuron developmental biology to provide some context for reviewing recent studies interrogating pathways that influence the generation of MN diversity. I conclude that motor neurogenesis from PSCs provides a powerful reductionist model system to gain insight into the developmental logic of MN subtype diversification and serves more broadly as a leading exemplar of potential strategies to resolve the molecular basis of neuronal subclass differentiation within the nervous system. These studies will in turn permit greater mechanistic understanding of differential MN subtype vulnerability using *in vitro* human disease models.

## 1. Introduction

**Figure 1:**
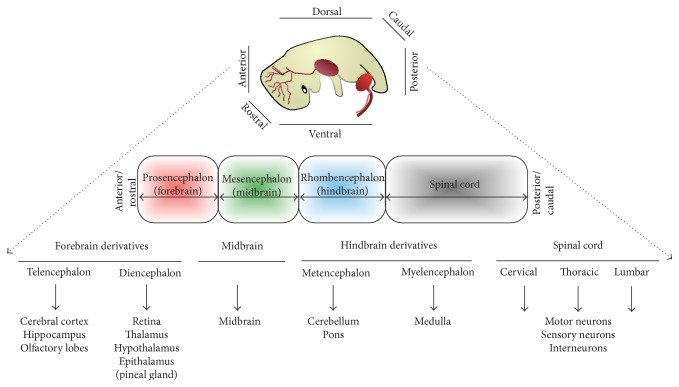
A simplified depiction of vertebrate nervous system regional organization (image of fetus adapted from [4]).

Human neurodegenerative disorders represent a spectrum of progressive and untreatable clinical diseases, characterized by selective loss of neurons, usually in a region-specific (e.g., Parkinson's disease) and/or subtype-specific (e.g., ALS) fashion. There is a great experimental need for renewable sources of clinically relevant, region-specific, and subtype-specific neurons. Lineage restriction and the generation of neuronal diversity within the developing neuraxis are consequences of the interplay of multiple developmental signals, which are regulated in a spatiotemporal manner. Precise cellular and molecular mechanisms through which these complex sequential and progressive developmental processes are orchestrated remain unresolved. The ability to generate defined neuronal cell types from PSCs offers a unique experimental opportunity to study the developmental mechanism(s) underlying generation of neural diversity during human embryogenesis [1, 2] (Figure 1). In turn, this will permit more accurate directed differentiation of regionally defined neurons for disease modeling, drug discovery, and potentially cell-based neural repair strategies.

Although often considered collectively as a group, neurons within an organism comprise highly diverse units differing in their gene expression profile, morphology, connectivity, functional characteristics, and response to injury or disease. Neuronal subtypes also differ markedly in developmental origin and anatomical location (Figure 1). Understanding how neuronal subtype diversity is accomplished within the developing neuraxis remains a major challenge in developmental neurobiology. Elucidating the transcriptional “logic” of cell fate specification is of equal relevance to the emerging discipline of regenerative neurology. The interconnectedness of developmental neurobiology and regenerative neurology is evident from global research efforts attempting to generate enriched populations of regionally defined and clinically relevant neuronal subtypes from PSCs. Such strategies for directed differentiation require an understanding of the embryonic origins of the neuronal subtype in question, allowing one to model neurodegenerative disease* in vitro* with fidelity and precision [3]. The fact that clinical neurodegenerative disease classically occurs in a region-specific and/or subtype-specific manner reinforces the importance of this line of enquiry. Selective vulnerability of individual subtypes of neurons underlies the majority of such progressive and incurable conditions. Against this background, spinal cord MNs provide a clinically relevant, prototypic example of cell fate specification, for which animal studies have already begun to elucidate the molecular basis of lineage restriction at specific developmental phases.

## 2. Motor Neuron Developmental Biology

Motor neuron specification requires several sequential developmental steps including neural induction from embryonic ectoderm, patterning along rostrocaudal and dorsoventral axes, and subsequently the terminal differentiation of regionally specified neural precursors into postmitotic neuronal subtypes. Following neural induction, precursors default to a rostral and dorsal positional identity through the combined actions of the BMP, WNT, and FGF signaling pathways, which have unique spatiotemporal influences on regional identity and cell fate [5–7]. Signaling pathways that operate along the rostrocaudal and dorsoventral neuraxes first establish a matrix of positional cues, which influence precursor cell fate specification by regulating the identities and concentrations of morphogenetic signals to which they are subjected [8].

### 2.1. Rostrocaudal (R-C) Patterning

**Figure 2:**
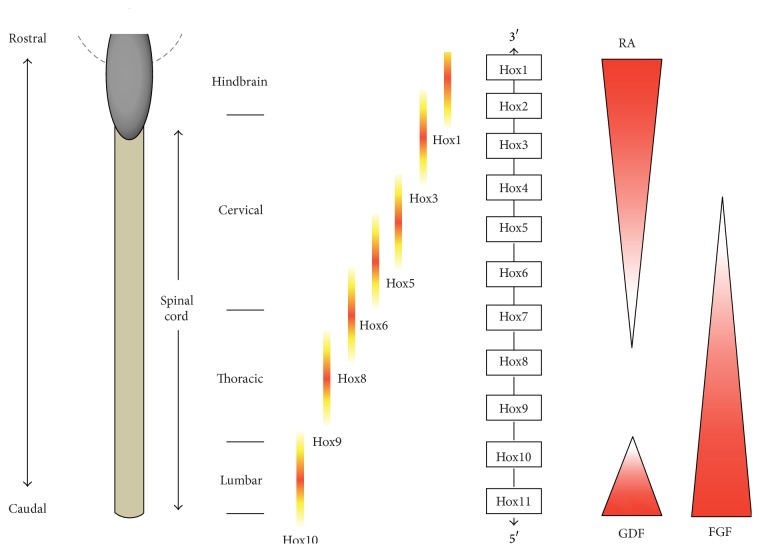
A schematic depicting expression patterns of chromosomally linked Hox genes along the rostrocaudal axis. Hox genes at one end of the cluster are expressed more rostrally, while those at the opposite end are expressed caudally. As indicated, RA is responsible for rostral spinal cord patterning (i.e., the cervical spinal cord). Caudal spinal areas are patterned by FGFs and GDFs. The precise nature of how these caudalizing factors conspire with one another has yet to be resolved. RA: retinoic acid; FGF: fibroblast growth factor; GDF: growth/differentiation factor.

Caudalizing morphogens respecify the positional identity of neurogenic precursors largely through their influence on the Hox genes, which are a family of transcription factors that regulate acquisition of positional identity in individual segments of the spinal cord [9, 10]. Hox genes contain a DNA sequence known as the “homeobox” and are further codified by their specific location in gene clusters within the genome, exhibiting R-C expression pattern that reflects their relative location within the gene cluster. Graded fibroblast growth factor (FGF) signaling functions along the R-C axis to induce the expression of chromosomally linked Hox genes in the neural tube. Hox genes located at one end of the cluster (3′ end) are expressed more rostrally in response to low levels of FGF; conversely, genes at the opposite end (5′ end) are expressed caudally in response to high levels of FGF. Different Hox “paralog” genes are consequently expressed at brachial (Hox4–Hox8), thoracic (Hox8-Hox9), and lumbar (Hox10–Hox13) levels of the spinal cord [11–13] (Figure 2). Body segmentation studies originated from work on the development of* Drosophila* body plan. In addition to determining the basic structure and orientation of an organism, Hox proteins also have a crucial role in determining the subtype diversification of MNs and their peripheral muscle target connectivity [14]. The mechanisms by which a Hox-based transcriptional network choreographs these processes are beginning to be resolved [15]. Retinoic acid (RA) and FGF signaling influence Hox expression in spinal cord neural precursors. Graded FGF signals regulate the primary Hox expression pattern before further superimposed cues refine subset-specific Hox gene expression. Rostrally, RA regulates Hox expression at cervical/brachial levels in part by antagonizing the FGF gradient [12, 16]. Caudally, Gdf11 (a member of TGF-*β* family) plays a critical role in Hox8–Hox10 gene expression at thoracic and lumbar spinal cord levels (Figure 2) [17, 18]. The mechanisms that translate graded signals into R-C positional information within the spinal cord remain largely unresolved. Indeed the temporal delay between Hox gene transcription and mRNA translation also remains elusive but this phenomenon may be at least partially accounted for by microRNA posttranscriptional regulation [19].

### 2.2. Dorsoventral (D-V) Patterning

**Figure 3:**
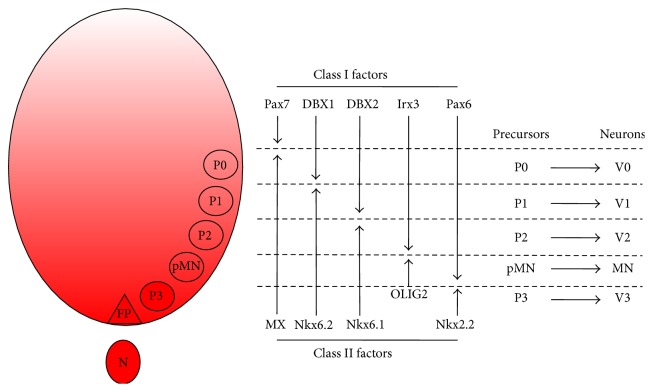
“Patterning” principles of the ventral neural tube by graded Sonic Hedgehog (Shh) signaling. Shh, shown in red, originates in the notochord (N) and floor plate (FP) and establishes a matrix of concentration-dependent positional identities, which are defined by particular combinatorial profiles of homeodomain transcription (HD) factors and basic helix-loop-helix (bHLH) transcription factors. Discrete ventral precursor domains are established by Shh-regulated transcription factor expression and divided into class I and class II factors based on their mode of regulation by Shh. These transcription factors are intermediaries in Shh-dependent ventral neural patterning. Cross-repressive interactions between class I and class II factors contribute to establishing individual precursor domains (P0–P3) with distinct boundaries as depicted. Each precursor domain gives rise to a specific class of postmitotic neurons. The cross-repressive class II factor counteracting Pax7 remains unresolved. Motor neurons are generated from the pMN domain.

During spinal cord development, several distinct neuronal subtypes are generated by the interaction of opposing morphogenetic gradients along the D-V axis of the neural tube, which establish a matrix of positional identities that in turn permit discrete precursor domains to emerge (Figure 3). This process underlies motor neurogenesis and ventral interneurogenesis [8]. Each neuronal group arises from discrete regions or “precursor domains” that are anatomically positioned in a stereotyped D-V arrangement. Ventral neuronal patterning results from morphogenetic cues emanating from a group of cells positioned at the ventral midline of the neural tube (called the floor plate) and the notochord [20]. In the early 90s, several labs successfully cloned vertebrate homologues of the* Drosophila* gene Hedgehog, which encodes secreted signaling proteins [21–24]. Sonic Hedgehog (Shh) was later discovered to be the ventrally secreted morphogenetic cue that confers D-V polarity to the ventral neural tube. Additionally, early experimental data suggested that Shh function was concentration-dependent [25]. Shh is a secreted glycoprotein expressed by the notochord and later by the floor plate, likely secondary to autoinduction [24, 26]. Motor neuron generation depends on two critical temporally distinct phases of Shh signaling: an early period, where it induces neural plate precursor cells to become ventralized, and a late period, where Shh drives the differentiation of ventralized precursors into motor neurons, at which point there is a concentration-dependent specification of ventral precursors into motor neurons or interneurons [27]. Antibodies against Shh inhibit motor neurogenesis in neural plate explants, despite the normal induction of floor plate cells under these conditions [25]. Key studies using animal explant cultures have defined important roles for Shh in floor plate specification, motor neurogenesis, and ventral spinal cord interneurogenesis [27]. These studies suggest that neural precursor cells require* early* exposure to Shh derived from the notochord soon after neural plate formation to permit competence for motor neurogenesis (Figure 3). The downregulation of Pax7 and Pax3 by neural plate cells temporally coincides with their competence to generate ventral cell types [28, 29]. The second and late critical period for Shh signaling in motor neurogenesis occurs after ventralization of precursors.* In vitro* studies using animal explants show that ventralized precursors deprived of Shh signaling cannot generate motor neurons [27]. Ventralized motor neuron precursors require Shh well into S phase of the final division cycle. The late dependence on Shh suggests that motor neuron identity is determined within late S phase of the final precursor cell division, consistent with studies addressing mammalian cortical laminar determination [30].

Shh signaling therefore determines neuronal subtype identity during the final division of ventral precursor cells, with higher concentrations promoting motor neuron specification and lower concentrations diverting cell fate to Lim1/Lim2 expressing interneurons [27]. The extent to which this implicates a common precursor has yet to be resolved. These studies show that the Shh concentration required to permit competence of neural plate cells for motor neurogenesis is 3 times lower than the concentration for the specification of motor neuron fate at a later stage. Furthermore, the Shh concentration for floor plate specification is 3 times greater than that for motor neuron generation [26]. Taken together, this suggests that different Shh concentrations operate at sequential periods during the generation of a single neuronal cell type and support the more generic concept that Shh controls the identity and pattern of ventral neural tube cell types by actions at multiple concentration thresholds.

The absolute requirement for Shh in floor plate and motor neuron differentiation, however, remains unresolved. Shh secreted from the notochord might therefore control patterning in the ventral neural tube entirely through local signaling, with long-range influences mediated by a secondary diffusible factor. Indeed, many of the long-range patterning activities of Hedgehog in* Drosophila* depend on the induction of diffusible intermediary factors, notably the transforming growth factor-*β*- (TGF-*β*-) like protein Decapentaplegic (DPP) [31, 32]. The necessity of Shh for motor neurogenesis and ventral interneurogenesis* in vivo* is supported by loss-of-function studies [27, 33]. Experiments using explants of naive neural tissue confirmed that varying concentrations of Shh protein specified distinct neuronal subtypes [34, 35].

These studies raise the issue of how positional identity is imposed on precursor cells and how this determines neuronal subtype identity. Several studies have suggested that a group of transcription factors, predominantly the homeodomain (HD) factors, are critical intermediaries in the process [34–37]. These transcription factors are expressed in stereotypic patterns along the D-V axis of the neural tube. Determined by their mode of regulation in response to Shh signaling, individual factors are designated as class I or II proteins. Class I proteins are repressed at particular concentrations of Shh, which thus defines their ventral limit of expression. Conversely, class II protein expression is induced by Shh, which therefore defines dorsal boundaries of their expression. Combinatorial expression of both classes of protein allows the establishment of five ventral neural tube precursor domains, which in turn permit the specification of distinct neuronal subtypes (Figure 3). The profile of HD protein expression thus appears to represent a transcriptional code that allocates positional identity to precursors, enabling differential neuronal subtype specification. Subsequent gain- and loss-of-function experiments in chick and mouse embryos have indeed provided further support for this putative mechanism, where ectopic expression of HD proteins predictably changed the position in which individual neuronal subtypes were generated in the neural tube [34–39]. Selective cross-repressive interactions between pairs of class I and II proteins expressed in adjacent precursor domains have since emerged as an important feature in the developmental logic of ventral spinal neurogenesis [34–37]. This principle of cross-repressive interactions observed in the neural tube is reminiscent of mechanisms involved in R-C patterning of the* Drosophila* embryo [40]. This raises the possibility that such processes may represent a more generic strategy underpinning the developmental logic for regional allocation of cell fate in response to morphogenetic instruction.

As described above, MNs originate from highly restricted foci in the ventral neural tube (pMN domain) in response to the morphogen Shh. In turn, Shh induces upregulation of the basic helix-loop-helix (bHLH) transcription factor Olig2, which then associates with another bHLH transcription factor, neurogenin2 (Ngn2), to direct the expression of MN fate consolidating genes such as Hb9 and Islet1 (Isl1) [41]. Olig2 is necessary for specification of MNs and, later in development, oligodendrocyte precursors. MN precursors are characterized by a particular “signature” of HD proteins and bHLH transcription factors including Pax6 and Olig2 as depicted in Figure 3 [34]. Each of these factors has a distinct role in MN specification. Pax6, for example, is involved in the establishment of discrete precursor domains in the ventral spinal cord and regulates cell fate specification of both motor neurons and interneurons via graded Shh signaling [35]. The combinatorial action of these HD proteins and bHLH transcription factors directs precursors to a terminally differentiated postmitotic state, after which they begin to upregulate fate consolidating downstream genes such as Hb9 [42–44]. The pMN domain precursors exclude Pax7, Irx3, and Nkx2.2 (Figure 3). If Shh is not administered in conjunction with RA, the resulting precursors fail to express pMN domain markers as they contain a mixed (dorsal to ventral) array of spinal cord regional identities. The degree to which precursors are ventralized in the spinal cord, which is dependent on the concentration of Shh used, is critical to cell fate determination and can be discriminated based on HD and bHLH factor expression profiles.

### 2.3. Motor Columns and Subtypes

**Figure 4:**
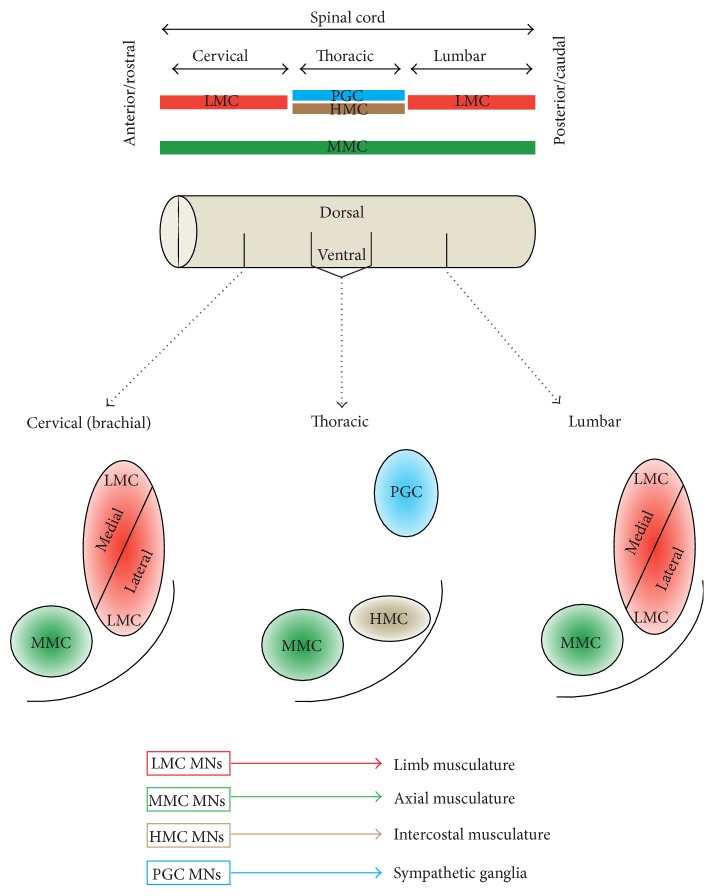
A schematic depicting columnar arrangement of motor neuron subtypes in relation to different rostrocaudal portions of the neuraxis. The LMCs exist only at cervical (brachial) and lumbar foci and are responsible for innervating the muscles of the upper and lower limbs, respectively. The MMC exists throughout the rostrocaudal extent of the spinal cord and is responsible for innervating axial musculature. The HMC and PGC exist only in thoracic portions of the spinal cord and innervate the intercostal musculature and sympathetic ganglia, respectively. R-C: rostrocaudal; LMC: lateral motor column; MMC: medial motor column; HMC: hypaxial motor column; PGC: preganglionic motor column; MN: motor neuron.

MN subclasses are further organized into groups that reflect both their developmental origins and also their adult function. Specifically, MNs are developmentally allocated to discrete motor columns, which extend along the R-C neural tube. Within a column, the group of MNs responsible for innervating a single skeletal muscle is termed a motor pool, each of which is also arranged by an anatomical logic corresponding to the muscle target(s) of its projections. The medial motor column (MMC) contains MNs that innervate dorsal epaxial muscles, which mainly subserve postural functions. MNs of hypaxial motor column (HMC) project to the ventral hypaxial muscles, which are involved in respiration. The lateral motor columns (LMCs) are responsible for innervating limb muscles. The preganglionic motor column (PGC) is present at thoracic levels and innervates sympathetic ganglia. The MMCs run throughout the R-C extent of the spinal cord, while the LMCs, HMCs, and PGCs occur only at brachiolumbar (LMCs) and thoracic (HMCs and PGCs) foci (Figure 4).

The molecular effectors that integrate morphogenetic extrinsic signals with transcription factor expression/repression to regulate neuronal subtype determination have been extensively studied* in vivo* [45–47]. Retinoid signaling plays key roles in the diversification of MN subtypes from the common MN precursor pool and additionally contributes to spinal cord columnar organization. These distinct MN subtypes can be discriminated based upon their gene expression profiles [48, 49]. At brachiolumbar foci, within the LMCs, RA is synthesized locally by subpopulations of MNs expressing RALDH2 and specifies migrating precursors into a distinct subset of LMC MNs (i.e., the lateral LMC MNs). The role of retinoid signaling in MN subtype specification has been demonstrated using heterotopic transplantation of RA synthesis “hotspots” (brachial and lumbar neural tube/somites) with subsequent characterization of regional MN subtype [50]. Additionally, ectopic RALDH2 expression in spinal neurons generates LMC MNs and RALDH2 knock-down and knock-out studies yield a reduction of both lateral and medial LMC neurons [49, 51]. These* in vivo* experiments also demonstrated marked depletion of dorsomedially positioned Isl^+^ preganglionic autonomic MNs (termed Column of Terni (CT) neurons in the chick [52]) upon expression of a constitutively active retinoid receptor in postmitotic MNs [49]. A significant reduction in ventrally positioned Isl1/2^+^, Lim3^−^ lateral MMC-like MNs was also found under these conditions [49]. However, inhibition of RA signaling in brachial motor neurons did not promote the differentiation of medial MMC neurons [49]. Interestingly, previous* in vivo* studies have demonstrated a regionally restricted role for retinoid signaling in the* postmitotic* specification of motor neuronal columnar identity [53, 54]. It is likely that the developmental programmes determining specification of distinct subtypes of MN require extrinsic morphogenetic instruction during both precursor specification and terminal differentiation. These studies illustrate the distinct requirements for RA signaling in MN generation and organization.

There is an unambiguous relationship between Hox protein expression and MN subtype determination. Specifically, Hox6 expression occurs in brachial LMC neurons, Hox9 proteins are expressed in thoracic PGC neurons, and Hox10 proteins are characteristically expressed by lumbar LMC neurons (Figure 2) [12, 13]. Cross-repressive, regulatory interactions occurring between these Hox proteins further refine expression profiles and facilitate MN subtype diversification. Misexpression experiments have suggested that Hox protein expression is sufficient to direct changes in MN columnar identity [13, 55]. The emerging logic of Hox functions along the R-C axis is reminiscent of mechanisms regulating D-V patterning (discussed above). In both cases, morphogenetic cues establish zones of homeodomain (HD) protein expression with subsequent domain refinement via selective cross-repressive interactions. Transcriptional cross-repressive programmes operate at temporally discrete stages of neurogenesis in each axis; they occur within neural precursor cells in the D-V axis [36] and in postmitotic neurons in the R-C axis [13]. The precise choreography of these sequential processes enables confinement of specific Hox codes to postmitotic MNs. Although hierarchical patterns in Hox expression have been posited in particular contexts [56], this does not appear to be a generic regulatory principle of Hox function [57, 58]. Functional dominances in Hox expression are likely spatiotemporally determined.

Experiments involving intercolumnar fate switching have demonstrated a consequent and congruent change in axonal trajection. For example, ectopic expression of Hoxc10 (lumbar) at thoracic levels causes a fate change to LMC MNs, which then project into the lower limb [55]. These experiments and others show similar results using Hoxc6 ectopic expression [59], thus confirming an additional role of Hox proteins in regulating peripheral connectivity of MNs to their targets. Although Hox proteins clearly have a critical role in MN subtype diversification, there is accumulating evidence that additional factors are necessary to refine their functions. Recognition of broad Hox expression throughout the embryo and developing CNS, combined with the observation that the same Hox protein is often expressed by multiple neuronal types within a given spinal cord segment, argues for the presence of additional regulatory factors. The existence of a hierarchy of Hox regulatory factors, including both generic and cell-type specific regulators, is supported by several lines of evidence [59–64]. A critical Hox cofactor regulating MN subtype diversity that has emerged from these studies is Foxp1, which appears to control columnar fate depending on its level of expression within individual MNs [14]. The function of Foxp1 has definitively been shown to be necessary for specification of both the LMC and PGC MN columns by genetic inactivation studies [14, 65]. Studies have also demonstrated that extrinsic signaling* postmitotically* is a key determinant of neuronal subtype diversification [8, 53, 54]. By utilizing a promoter that directs transgene expression to postmitotic neurons, the timing of retinoid mediated MN subtype diversification has been studied [42, 54]. Such experiments definitively show that MN subtype identity remains plastic after cell cycle exit [49]. Experimental alteration of HD proteins within postmitotic MNs has also been shown to result in subtype switching [54].

Against the background of these studies, it is clear that the descriptive term “MN” is an oversimplification and the numerous motor neuronal subtype differences described above (including R-C position, column, pool, and axonal trajection) begin to demonstrate some of the necessary complexity inherent in MN diversification. The developmental biology that underpins MN differentiation is relatively well understood and thus provides a rational basis for using human stem cell-based systems to allow further elucidation of mechanisms responsible for generating MN subtype diversity.

## 3. Pluripotent Stem Cells

There are different sources of species-specific stem cells including embryonic, fetal, and adult varieties. Embryonic stem cells (ESCs) are a type of PSC thus possessing the greatest developmental potential* in vitro.* PSCs respond predictably to developmental cues such that their fate can be systematically manipulated to differentiate into myriad cell lineages from any of the 3 germ layers. There are 3 principal properties that characterize PSCs: (i) the ability to self-renew, (ii) pluripotency (i.e., the ability to specialize into any of the cell types that comprise the organism from which they are derived) [66], and (iii) chimera formation. Optimization of appropriate* in vitro* culture conditions for the propagation of undifferentiated vertebrate tumor cell lines in the 1970s [67] heralded the isolation of mouse ESCs in 1981. The key role of leukemia inhibitory factor in maintaining pluripotency was later demonstrated [68]. Human ESCs were first isolated in 1998 [69], some 17 years after the isolation of mouse ESCs [70]. Human ESCs are isolated from the inner cell mass of a preimplantation embryo, from where cells are micromanipulated and subsequently grown in culture by a variety of standardized methods [71–73]. Human ESCs can be reliably identified by the expression of well characterized transcription factors and surface antigens [74]. It is noteworthy that the blastocysts from which human ESCs are derived exist in surplus and are donated to research by individuals under fully informed consent at* in vitro* fertilization clinics [75–77]. Establishment of optimal culture systems for mouse ESCs guided human PSC culture techniques. However, significant differences exist including the signaling pathways required for maintaining pluripotency, FGF, Activin/Nodal, and WNT being more important in human PSCs than their mouse counterparts [78–80]. Feeder-free human PSC culture systems, among several other innovations to the culture constituents, have provided optimism for establishing clinical-grade cells for use in regenerative medicine [73]. These refinements have somewhat addressed early concerns around potential xenoinfection [81].

Compared to PSCs, stem cells isolated at later developmental stages possess a restricted phenotypic potential that is tissue specific. Regional restriction (e.g., forebrain versus spinal precursors) also represents a potential problem because this cannot, at present, be predictably altered using extrinsic signals. This tissue specific “multipotency” (i.e., the ability to differentiate into some but not all cell types that comprise an organism) is a relative disadvantage when studying cell fate specification, where the pluripotent state represents an ideal investigative tool. Attempts to derive PSCs by different methods have partially tempered the ethical controversy in which the human ESC field is immersed [82]. These efforts were largely inspired by earlier seminal work from Gurdon and Wilmut [83, 84] and focus on nuclear reprogramming by somatic cell nuclear transfer (SCNT) and cell fusion techniques [85]. Both methods, however, have significant drawbacks: the requirement of oocytes in the former and tetraploidy in the latter. Subsequent efforts have also achieved ESC derivation from single blastomeres [86]. This area of research culminated in the discovery of induced pluripotent stem cells (iPSCs), where adult somatic cells are reprogrammed to embryonic-like stem cells by the viral transduction of four transcription factors [87]. Other studies have also achieved transcriptional or “forward” programming where precursor cells are essentially converted to a desired cell type using defined factors [88]. A further significant advance that has been made is that of “transdifferentiation” of one differentiated cell type into another. One such study reprogrammed fibroblasts directly into functional neurons [89] using defined virally transduced genes. Indeed, another recent report describes the direct conversion of mouse and human fibroblasts into functional* and regionally defined* dopaminergic neurons [90]. These discoveries represent a paradigm shift in the current thinking of lineage restriction.

## 4. Directed Differentiation of PSCs to Motor Neurons

Predictable and scalable directed differentiation of PSCs to the neural lineage is necessary for studying human neural development, modeling disease, and drug discovery. Several well established methods can robustly achieve neural conversion of PSCs in chemically defined conditions [91–98]. Efficient neural conversion in chemically defined medium is based on the default model of neurogenesis [99, 100] where extrinsic and intrinsic signals that could divert differentiation to alternate fates are minimized in culture. Inhibition of Activin/Nodal signaling accelerates neural conversion from PSCs [101] and additionally imposes a caudal positional identity on resulting precursors [102]. Inhibition of both the Activin/Nodal and BMP arms of the TGF-*β* signaling superfamily permits highly efficient neural conversion of PSCs and represents the most widely adopted method employed to date [92].

The elucidation of developmental inductive cues and transcriptional programmes for MN specification [8] have been instrumental in the directed differentiation of PSCs. By recapitulating a developmentally rationalized programme of morphogenetic cues, reproducible MN differentiation has been demonstrated from mESCs [103] and human ESCs [1]. Such studies confirm the expression of MN associated transcription factors including Isl1 and Hb9. The detection of specific enzymes/transporters including choline acetyltransferase (ChAT) and the vesicular acetylcholine neurotransmitter transporter (vAChT) provides further validation. Additionally, we and others have employed coculture methods with myotubes to demonstrate the formation of physiologically relevant neuromuscular junctions by human PSC-derived spinal MNs [1, 104, 105]. Importantly, electrophysiological studies confirm that motor neurons differentiated from mESCs (and later human PSCs) acquire appropriate functional properties [104]. Furthermore, these motor neuron precursors survive and integrate in rodent embryonic spinal cord [103, 106] and extend axons forming physiologically relevant synapses. Functional engraftment has also recently been demonstrated following peripheral motor nerve transection [104]. Taken together, these studies convincingly demonstrate functional motor neurogenesis from human PSCs.

Insights from developmental studies across different species and from* in vitro* work using human PSCs suggest that, soon after neural induction, precursors initially assume a rostral and dorsal positional identity through the combined actions of the BMP, WNT, and FGF signaling pathways, which have unique spatiotemporal influences on positional identity and cell fate decisions [5, 6]. Mouse and human PSC-derived neural precursors can differentiate into MNs by application of a programme of extrinsic signals (FGF-2, retinoic acid, and Sonic Hedgehog (Shh)) that recapitulate the developmental process of neural patterning (discussed above)* in vitro* [1, 103]. Once neural precursors have been positionally specified to the ventral spinal cord, they can be plated down for terminal differentiation. This usually involves a poly-D-lysine/laminin substrate with concomitant withdrawal of mitogens and addition of neurotrophic factors. Using this approach in an adherent culture system, Olig2 precursors first appear at 2-3 weeks and markers of mature MNs (such as HB9) appear at 4-5 weeks [1].

Important future studies would include systematic analyses of the requisite time for neural patterning prior to terminal differentiation, which has yet to be definitively addressed. Some studies have suggested that accelerated neural conversion and patterning protocols are possible. The question of sequential* versus* simultaneous administration of morphogenetic signals is another interesting subject, which merits discussion here. In the case of motor neurons, for example, the first directed differentiation of MNs from mouse ESCs employed simultaneous administration of RA and Shh [103], whereas when this was first achieved in human PSCs [1] the cues were introduced sequentially (RA before Shh). Given the complex spatiotemporal regulation and reiterative use of such canonical signaling pathways during embryogenesis, their individual and combinatorial applications at both earlier and later time points of currently established differentiation protocols are worthy of consideration. Such studies will help to uncover mechanisms underlying the generation of neuronal diversity by more closely approximating* in vivo* motor neuron lineage restriction.

## 5. Generating Diverse MN Subtypes from Human PSCs

There is an increasing wealth of literature around the mechanisms underlying neurodevelopment, revealing a remarkable and previously unrecognized extent of neuronal diversity. Functionally relevant differences in molecular phenotype, axonal projection, dendritic arborization, and electrophysiological attributes have been demonstrated between different neuronal subtypes throughout the neuraxis [107–110]. The ability to generate defined cellular subtypes from human PSCs through application of neurodevelopmental principles offers a unique experimental opportunity to interrogate the molecular mechanisms underlying human neuronal diversity [1, 2].

To date, the vast majority of studies using ESCs have focused on deriving MNs generically, while comparatively few studies address the issue of motor neuronal subtype diversification. Most protocols for motor neuron specification from PSCs (either ESCs or iPSCs) utilize the application of a simplistic programme of morphogenetic signals to achieve neural patterning, followed by standard terminal differentiation conditions. Signaling mechanisms and transcriptional events involved in the postmitotic diversification of motor neuronal subtypes from human ESCs remain poorly characterized. Currently, no study has systematically investigated the influence of a morphogenetic signal during both MN precursor “patterning” and terminal differentiation in order to establish their relative contributions to subtype choice during these distinct developmental stages.

MNs are a diverse collection of neuronal subtypes displaying differential vulnerability in different human neurodegenerative diseases. During embryogenesis, retinoid signaling contributes to caudal precursor specification generically and subsequent MN subtype diversification. RA typically results in a cervical or brachial positional identity [105, 106]. Against this background, we reported a retinoid independent strategy for generating MNs from human PSCs that yields a predominantly caudal (lumbar spinal) MN identity and bias to the MMC (both lateral and medial subdivisions) [105]. Recognition that subclasses of MNs can be specified independently of RA in the context of Activin/Nodal inhibition and Hedgehog signaling increases the potential diversity of human motor neurons for study. More caudal MN fates can be achieved in the absence of RA signaling (possibly in response to FGF2). In addition, RA-independent motor neurogenesis results in a MMC subtype bias compatible with the known contribution of RA to subtype determination [50]. Recent evidence suggests a role for Hoxc9 as a “master regulator” of motor system organization through global cross-repressive activities [15]. Indeed our own data suggest significantly more induction of Hoxc9 in RA-treated when compared to RA-independent cultures (although expression is evident in both conditions). Further manipulation of Hoxc9 expression, via extrinsic signals or gene targeting, may facilitate more refined approaches to directed differentiation of human PSCs to enriched populations of motor neuronal subtypes. Foxp1 (high) (LMC) human PSC-MNs are specified with continued retinoid exposure, while inhibition of this pathway in MNs is shown to divert LMC subtype to a lateral MMC (or HMC) identity, consistent with previous developmental* in vivo* studies [48]. Therefore, these findings support a model whereby retinoid signaling promotes the specification of LMC MNs at the expense of lateral MMC MNs. Furthermore, the refractoriness of the medial MMC to changes in retinoid signaling implicates its development as a motor neuronal “ground state.” This retinoid-mediated diversification of motor neuron subtypes was also supported by a contemporaneous study using mouse ESCs [106]. Separately, a recent report described combining retinoic acid and a WNT agonist to generate cranial motor neurons from human PSCs [111]. Collectively, these studies support the view that the retinoid pathway plays key roles in subtype determination within the MN pool [48, 49, 111–113]. More subtle methods of generating MN subtype diversity include manipulating the concentration or chemical composition of extrinsic cues. This strategy was recently employed by a study substituting recombinant Shh for SAG (a chlorobenzothiophene-containing Shh pathway agonist) and purmorphamine [114] (a small-molecule agonist for Smoothened) in combination, with consequent subtype variation of MMC versus LMC MNs [115].

Separately, the use of suspension culture [102, 105, 106, 115, 116] versus adherent culture methods [92, 117] by different groups will likely have a significant impact on the subtype diversity of MNs generated and requires further study. Transcriptional or “forward” programming approaches [118], in addition to improving the efficiency and timing of directed differentiation of PSCs, have also yielded some relevant insights into mechanisms underlying MN subtype determination. In mouse ESCs, three transcriptional (forward programming) factors, Ngn2, Isl1, and Lhx3, were sufficient to specify spinal MN identity. In this study, replacing the Lhx3 programming factor with Phox2a yielded cranial rather than spinal MNs [119]. Transdifferentiation [89] approaches have also successfully generated MNs from human PSCs [120], providing an ideal “test-bed” for elucidating the transcriptional logic of MN subtype determination. The functional implications of these studies are of considerable interest because motor column organization in development forms the basis for motor pools and target innervation [121]. Furthermore, in diseases such as ALS, MN subtypes exhibit differential vulnerability to degeneration.

## 6. Conclusions

Elucidating efficient protocols to generate enriched populations of MN subtypes has important biotechnological implications for disease modeling, drug discovery, and potentially cell-based therapy. Such approaches would permit comprehensive mechanistic studies of differential MN subtype vulnerability* in vitro.* Access to human PSCs allows questions around early cell fate acquisition to be addressed. Developmental competence to extrinsic morphogenetic signals during embryonic patterning is both spatially and temporally restricted. Human PSCs faithfully recapitulate the key milestones of neurodevelopment and thus permit studies that will improve our understanding of the relative contributions of extrinsic signals, cell-intrinsic transcriptional programmes, and intercellular/paracrine communication to direct cell fate decisions. This reductionist model system, together with the use of a fully defined cell culture medium, provides a unique experimental opportunity to understand the relevance of how signaling pathways function individually or combinatorially to direct lineage restriction at different developmental stages. In turn, such knowledge will permit more predictable manipulation of PSCs to generate desired progeny for experimental study. PSCs therefore represent an unparalleled opportunity to study human neurodevelopmental processes. It is likely that, for future strategies aimed at generating distinct subtypes of MN, controlled extrinsic morphogenetic instruction during both precursor specification and terminal differentiation will be key determinants of success. As a final remark, although there is a considerable degree of conservation in neuraxial systems between rodents and humans, important differences in architecture, computational power, and functional capacity exist. Such evolutionary divergence strongly reinforces the indispensability of human experimental systems to complement,* but not replace*, existing rodent-based approaches.
